# Fear of infection and optimism predict following stay-at-home recommendations during COVID-19 pandemic in russian young people

**DOI:** 10.1192/j.eurpsy.2021.288

**Published:** 2021-08-13

**Authors:** T. Gordeeva, O. Sychev, O. Vindeker

**Affiliations:** 1 Research Department, Shukshin Altai State University for Humanities and Pedagogy, Moscow, Russian Federation; 2 Psychology, National Research University Higher School of Economics and Department of Psychology Moscow State University, Moscow, Russian Federation

**Keywords:** Fear of infection, Defensive optimism, COVID-19 pandemic, stay-at-home recommendations

## Abstract

**Introduction:**

. Self-isolation regime is an effective measure to contain the pandemic (Alfano, Ercolano, 2020), but the psychological factors predicting compliance with stay-at-home recommendations (CSHR) are understudied. We hypothesized that 1) defensive optimism and constructive optimism will have opposite effects on CSHR, 2) the effect of defensive optimism will be mediated through a decrease of anxiety (fear of infection).

**Objectives:**

. The purpose of this study was to assess the direct and indirect (through the fear of infection) effects of defensive optimism (belief that coronavirus problem is exaggerated) and constructive optimism (belief that people’s efforts help to prevent infection and spread of the virus) on CSHR, controlling for dispositional optimism.

**Methods:**

. A longitudinal study (from 10/4/2020 till 2/6/2020) was conducted on a sample of 306 university students (89% women, MA=21.20, SD=4.54) using a single-item measure of CSHR, LOT-R (Scheier et al., 1994), the scales of defensive and constructive optimism (Gordeeva, Sychev, 2020), and anxiety in a pandemic situation questionnaire (Tkhostov, Rasskazova, 2020).

**Results:**

. During seven-week interval CSHR has decreased dramatically (Cohen’s d=0.66, p<0.001) while the other variables remained stable. Using SEM we have showed that CSHR at the end of study (T2) is predicted by the CSHR (T1) and through it by the defensive optimism (negative effect, p<0.05) and constructive optimism (positive effect, p<0.001). Negative effect of defensive optimism on CSHR is also mediated by the fear of infection (T2), reducing it. Dispositional optimism is associated only with constructive optimism.

**Conclusions:**

. Defensive and constructive optimism/ pessimism are essential in explaining health-related behavior.

**Disclosure:**

No significant relationships.

